# Distinct *Aeromonas* Populations in Water Column and Associated with Copepods from Estuarine Environment (Seine, France)

**DOI:** 10.3389/fmicb.2017.01259

**Published:** 2017-07-11

**Authors:** Gautier Chaix, Frédéric Roger, Thierry Berthe, Brigitte Lamy, Estelle Jumas-Bilak, Robert Lafite, Joëlle Forget-Leray, Fabienne Petit

**Affiliations:** ^1^UNIROUEN, UNICAEN, CNRS, M2C, Normandie Université Rouen, France; ^2^CNRS 5569 HSM, Équipe Pathogènes Hydriques Santé Environnements, Université de Montpellier Montpellier, France; ^3^Laboratoire de Bactériologie, CHU de Nice Nice, France; ^4^Département d’Hygiène Hospitalière, CHRU de Montpellier Montpellier, France; ^5^ULH UMR I-02 SEBIO, FR CNRS SCALE, Normandie Université Le Havre, France; ^6^UPMC, CNRS, EPHE, UMR 7619 METIS, Sorbonne Universités Paris, France

**Keywords:** *Aeromonas*, diversity, copepods, estuary, antibiotic resistance

## Abstract

*Aeromonas* spp. are ubiquitous bacteria primarily recovered from aquatic ecosystems. They are found in fresh water as well as estuarine and marine waters, and in association with numerous autochthonous aquatic organisms in these environments. However, aeromonads are also etiologic agents of fish diseases and are now recognized as emerging pathogens in humans. The estuary is therefore a key environment, harboring autochthonous aeromonads, and aeromonads originating from humans and animals, mainly released by treated WWTP effluent or watershed run-off via tributaries. The present study compares the abundance and the diversity of *Aeromonas* populations. Over 2 years of monitoring (eight campaigns from February 2013 to November 2015), the occurrence of *Aeromonas* was investigated within the water column (water and fluid mud) and in association with copepods. Moreover, the diversity of *Aeromonas* populations was ascertained by analyzing *gyrB* and *radA* sequences, and the antibiotic-resistance phenotypes were determined using the disk diffusion method. This study shows, for the first time, the presence of *Aeromonas* spp. in water (1.1 × 10^2^ to 1.2 ± 0.3 × 10^3^ CFU.100 mL^-1^), fluid mud (2.6 ± 2.6 × 10^2^ to 9.8 ± 0.9 × 10^3^ CFU.g^-1^) and in association with living copepods (1.9 ± 0.7 × 10^2^ to >1.1 × 10^4^ CFU.g^-1^) in the Seine estuary. Moreover, the diversity study, conducted on 36 strains isolated from the water column and 47 strains isolated from copepods, indicates distinct populations within these two compartments. Strains distributed in five clusters corresponding to *A. bestiarum* (*n* = 6; 5.45%), *A. encheleia* (*n* = 1; 0.91%), *A. media* (*n* = 22; 20.0%), *A. rivipollensis* (*n* = 34; 30.91%) and *A. salmonicida* (*n* = 47; 42.73%). *A. salmonicida* is the most abundant species associated with *Eurytemora affinis* (*n* = 35; 74.47%). In contrast, *A. salmonicida* accounts for only 30.56% (*n* = 11) of isolates in the water column. This study shows the coexistence of distinct populations of *Aeromonas* in the oligohaline area of an anthropized estuary. Moreover, *A. media*, a putative human pathogen, present in the water column and abundant in the WWTP samples, was not detected in association with living copepods.

## Introduction

*Aeromonas* spp. are ubiquitous bacteria primarily recovered from aquatic ecosystems (Janda and Abbott, 2010). They are found in fresh water as well as estuarine and marine waters, and in association with numerous autochthonous aquatic organisms in these environments (Gugliandolo et al., 2008; Janda and Abbott, 2010; Khor et al., 2015; Laviad and Halpern, 2016; Chenia and Duma, 2017). They are also isolated from various habitats such as the gastrointestinal tracts of healthy animals and as transient flora in foods such as vegetables, dairy products, meat, seafood, and drinking water.

Several species are involved in pathologic interaction with numerous animals from the aquatic environment, mainly fishes but also corals, for example (Janda and Abbott, 2010; Hamid et al., 2016; Chenia and Duma, 2017). Some of these species have a major economic impact in aquaculture: the species *A. hydrophila*, *A. caviae*, and *A. veronii* are frequent etiologic agents of fish diseases, e.g., motile *Aeromonas* septicemia and ulcerative syndrome; *A. salmonicida* is responsible for fish furunculosis, which can cause death within hours (Menanteau-Ledouble et al., 2016). In addition, the hypervirulent pathotype of *A. hydrophila* is considered an emerging pathogen responsible for outstanding epidemic outbreaks in farmed warm-water fishes (Rasmussen-Ivey et al., 2016).

Moreover, aeromonads are recognized as emerging pathogens in humans (Janda and Abbott, 2010). The severity of the disease varies from diarrhea to septicemia, depending on individual’s susceptibility, mainly the immune status (Aujoulat et al., 2012; Figueras and Beaz-Hidalgo, 2015; Teunis and Figueras, 2016). The species *A. hydrophila*, *A. veronii*, *A. caviae*, *A. dhakensis*, and *A. media* are responsible for more than 85% of human cases of aeromonosis (Figueras and Beaz-Hidalgo, 2015; Teunis and Figueras, 2016). Transmission of pathogenic strains of *Aeromonas*, including the fecal-oral route, is often due to direct or indirect contact with water (Lamy et al., 2009; Janda and Abbott, 2010; Li et al., 2015; Pal et al., 2016).

To date, widespread of antibiotic-resistant bacteria such as *Aeromonas* is a major public health issue related to the One Health concept, considering that aeromonads circulate within the major ecosystems: human, animals and water. Occurrence of antibiotic-resistant *Aeromonas* has been reported in these three ecosystems (Figueira et al., 2011; Piotrowska and Popowska, 2014; Esteve et al., 2015; Li et al., 2015; Patil et al., 2016; Baron et al., 2017). In this context, *Aeromonas* has been recently proposed as an indicator to assess the spread of antibiotic resistance in the aquatic environment (Berendonk et al., 2015).

Among water environments, estuaries are of particular interest for *Aeromonas* ecology. They are a transitional zone between a freshwater river and the seawater, characterized by a salinity gradient, where the level of contamination by chemicals and fecal bacteria reflect the land use of the watershed. In this environment, *Aeromonas* has been isolated in both water and sediments; some isolates exhibited resistance to multiple antibiotics (Henriques et al., 2006; Silva et al., 2014). Thus, in a lagoon estuarine area (Abidjan, Africa), the maximum abundance of *Aeromonas* was linked to both discharges from urban areas and the river-flow period when the water salinity was low (Marcel et al., 2002). In estuaries, aeromonads were reported to be associated with copepods, the most abundant zooplankton living in the salinity gradient zone and a key component in estuarine food chains. Interestingly, in coastal water in Italy, Gugliandolo et al. (2008) showed that abundance of *Aeromonas* spp. associated with the copepods is higher than in the water column, suggesting that copepods may function as an aeromonad reservoir. However, today little is known about the putative role of copepods as a vector of pathogenic strains for humans, and the population diversity of *Aeromonas* associated with copepods remains poorly documented. The topic is complicated by the fact that (i) there are still no simple culturable methods to recover *Aeromonas* from the environment (Latif-Eugenín et al., 2016) and (ii) the aeromonad population structure is a complex of species, making taxonomic classification difficult. Multilocus phylogenetic analysis (MLPA) and/or whole-genome sequencing are now used for a more precise delineation of the *Aeromonas* species (Alperi et al., 2010a; Roger et al., 2012; Colston et al., 2014; Talagrand-Reboul et al., 2017), with new species of *Aeromonas* autochthonous in water environments regularly described (e.g., Marti and Balcázar, 2015; Latif-Eugenín et al., 2016).

The Seine estuary is the largest macrotidal estuary opening into the English Channel, which is characterized by strong anthropic pressure exerted on its watershed (76,650 km^2^): 30% of the French population is located mainly in its urban areas, with 40% of the country’s economic activity (mostly the chemical industry) and 30% of the national agricultural activity. The estuarine water quality has been extensively studied within the framework of the French multidisciplinary scientific program^1^. The microbiological quality of the Seine estuary water is poor, mainly impacted by wastewater treatment plants (WWTPs) treating wastewater from Paris and its suburbs during high flow-periods, while the input of Seine tributaries are predominant during low-flow periods (Garcia-Armisen et al., 2005; Servais et al., 2007; Touron et al., 2007). In addition, high contamination by trace metals, mainly cadmium and lead, and organic compounds such as polycyclic aromatic hydrocarbons, (PAHs), polychlorinated biphenyls (PCBs) and pesticides make the Seine estuary one of the most contaminated in Europe (Carpentier et al., 2002).

This macrotidal estuary is characterized by the presence of a high turbidity zone (HTZ) in the lower estuary (in the mouth of the estuary) where suspended particulate matter (SPM) and the associated contaminants are concentrated. Therefore, in this area the behavior of microorganisms such as fecal bacteria, mainly associated with organomineral particles, is strongly influenced by hydrosedimentary processes (Guézennec et al., 1999; Pachepsky and Shelton, 2011; Malham et al., 2014). During a semidiurnal tidal cycle, bacteria associated with particles suspended in the water column settle during slack high water and then are concentrated in fluid mud at the water–sediment interface. In contrast, at the beginning of a flood, when current velocities increase, bacteria trapped within the fluid mud can be resuspended in the water column (Berthe et al., 2008). Another characteristic of this area is the abundance of *Eurytemora affinis*, a distinctly dominant copepod species (crustacean, calanoid). In this oligohaline zone, *Eurytemora affinis* accounts for up to more than 90% of the zooplankton (Mouny and Dauvin, 2002; Devreker et al., 2010). The dynamics of population of *Eurytemora affinis* was driven by environmental parameters (SPM, salinity and temperature) and consequently – as bacteria dynamic-closely linked to the tidal cycle (Devreker et al., 2010).

Thus, the mouth of Seine estuary is a key environment and provides an outstanding model for studying autochthonous aeromonads in the estuary, and those from human and animal origin, mainly released by treated effluent from WWTPs or watershed run-off via tributaries. The present study aims to compare the abundance and the diversity of *Aeromonas* populations (i) in the water column, i.e., water and fluid mud, (ii) in association with copepods and (iii) from treated effluent from WWTP released in the same area. For this purpose, a 2-year monitoring campaign was carried out in the Seine estuary, and the diversity of the *Aeromonas* population was investigated based on a combined culturable and molecular approach, and the phenotypic antibiotic-resistance profile was determined.

## Materials and Methods

### Sampling Strategy

**FIGURE 1:**
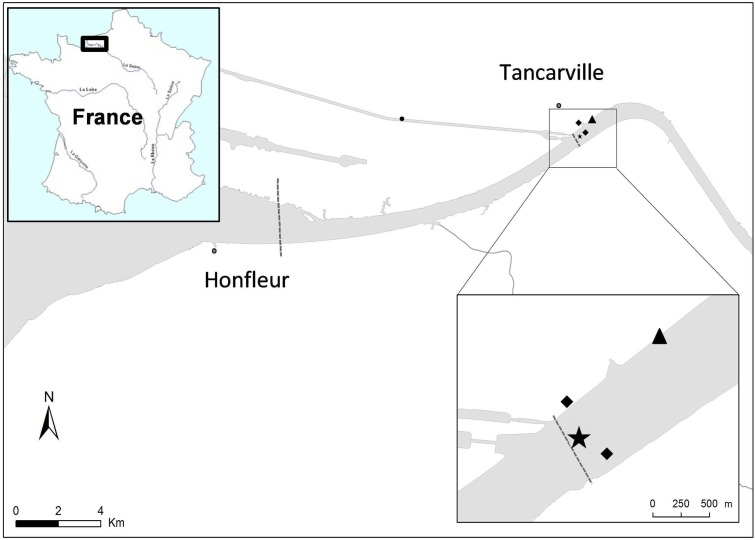
Study area and sampling point. Water, fluid mud and copepods were sampled from the mouth of Seine estuary (France N 49° 28′ 30.26″ E 0° 27′ 48.65″) located at 1.2 km downstream of a wastewater treatment plant (WWTP; 1800 inhabitants). Surface water (–50 cm) was sampled in the middle of the channel, fluid muds were sampled from the subtidal mudflat (February 2012, March, May 2013 and October 2015) and from the intertidal mudflat (August 2014, February, June and November 2015) and copepods were collected from the right bank (February 2012, March 2013 and October 2015). 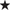, Water sampling point; 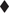, Fluid mud sampling points; 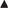, WWTP.

Copepods, water, and fluid mud were collected in the mouth of Seine estuary (France N 49° 28′ 30.26″ E 0° 27′ 48.65″) (**Figure 1**). This site is located in the mesohaline zone where salinity can range from 0 to 15 during a semidiurnal cycle (twice a day). In this area the microbiological quality based on *Escherichia coli* and *Enterococci* abundance ranged from 3.0 × 10^1^ to 2.5 × 10^3^ CFU.100 mL^-1^ and 1.0 × 10^1^ to 2.3 × 10^3^ CFU.100 mL^-1^, respectively (Touron et al., 2007), resulting in (i) intra-estuarine inputs (WWTP and seven main tributaries) and (ii) inputs of the estuarine entrance mainly dominated by one of the largest WWTPs in Europe, which treats the wastewater of Paris and its suburbs (6.5 million inhabitants). Moreover, a Waste Water Treatment Plan (Tancarville WWTP, 1800 inhabitants) is located 1 km upstream from the sampling site. Between February 2012 and November 2015, eight sampling campaigns were carried out at 0 and 2 h after the high water slack for various hydrological periods: in the high-flow period (>800 m^3^.s^-1^) and low-flow period (<500 m^3^.s^-1^, **Table 1**). The HTZ was located in this area for five of the eight campaigns (February 2012, August 2014, June 2015, October 2015, and November 2015). Surface water (-50 cm depth) and bottom water (+50 cm from the bottom) were sampled with a 3-L Niskin bottle and were transferred to sterile bottles before being analyzed. Fluid mud was sampled (i) on the intertidal mudflat (August 2014, February, June and November 2015) and directly collected with sterile plastic tubes at three equidistant points 50 cm apart; (ii) on the subtidal mudflat, closest to the copepod sample site (March and May 2013, and October 2015) with an Ekman sediment grab sampler (15 cm × 15 cm area). All the samples were immediately stored at 4–6°C after sampling and microbiological analysis was carried out within 4 h. Treated WWTP effluent (500 mL) (**Figure 1**) was collected every hour for 24 h using an ISCO 6700 portable sampler (Teledyne Isco, Inc., Lincoln, NE, United States). The mean daily sample consists of a subsamples mixture (1 flask of 1 L every hour) of identical volume (250 mL) collected during the sampling period (24 h).

**Table 1:** Hydrological, physicochemical, microbial, and biotic characteristics of the sampling site.

	February 2012		March 2013		May 2013		August 2014		February 2015		June 2015		October 2015		November 2015	
**Hydrological and physico-chemical parameters**					
Flow rate^a^	385 m^3^.s^-1^		877 m^3^.s^-1^		1090 m^3^.s^-1^		366 m^3^.s^-1^		1144 m^3^.s^-1^		226 m^3^.s^-1^		217 m^3^.s^-1^		199 m^3^.s^-1^	
Pluviometry^b^	2.8 mm		6.4 mm		35.4 mm		37 mm		20.7 mm		16.9 mm		13.4 mm		6.6 mm	
Salinity	1.3		0.2		0		0.6		0		8.3		5.2		2.8	
Temperature	7°C		7.2°C		15°C		19.5°C		5.4°C		19.8°C		15.4°C		12°C	
Conductivity	1784 μS.cm^-1^		784 μS.cm^-1^		NA		1400 μS.cm^-1^		477 μS.cm^-1^		13086 μS.cm^-1^		8896 μS.cm^-1^		4012 μS.cm^-1^	
SPM^c^	100 mg.L^-1^		50 mg.L^-1^		10 mg.L^-1^		68 mg.L^-1^		88 mg.L^-1^		76 mg.L^-1^		81 mg.L^-1^		185 mg.L^-1^	
**Microbiological quality**					
	Water^d^	Fluid mud^e^	Water	Fluid mud	Water	Fluid mud	Water	Fluid mud	Water	Fluid mud	Water	Fluid mud	Water	Fluid mud	Water	Fluid mud
*E. coli*	NA	NA	7.0 ± 0.2 × 10^1^	6.1 ± 0.7 × 10^0^	NA	5.5 ± 0.3 × 10^1^	1.1 ± 0.2 × 10^2^	2.1 ± 1.2 × 10^1^	3.0 ± 1.4 × 10^1^	3.5 ± 0.9 × 10^2^	6.2 ± 0.1 × 10^1^	<5.0 10^1f^	2.3 ± 0.4 × 10^2^	3.9 ± 1.6 × 10^1^	5.0 ± 1.0 × 10^1^	6.6 ± 2.0 × 10^0^
*Enterococci*	2.0 × 10^1^	NA	5.0 ± 0.5 × 10^1^	6.5 ± 0.3 × 10^0^	NA	5.7 ± 1.5 × 10^1^	2.5 ± 0.7 × 10^1^	5.0 ± 5.0 × 10^0^	2.6 ± 0.4 × 10^2^	5.2 ± 0.9 × 10^2^	<5.0 10^1f^	2.6 ± 0.5 × 10^1^	7.5 ± 3.5 × 10^1^	3.6 ± 0.4 × 10^1^	<5.0 × 10^1f^	8.0 ± 3.0 × 10^1^
**Copepods**																
*Eurytemora affinis*	+		+		-		+		NS		NS		+		NS	


*Surface water (-50 cm below the surface) and fluid mud were sampled in the mouth of the Seine Estuary at Tancarville site (N 49° 28′ 30.26″ E 0° 27′ 48.65″). ^a^Average daily flow rate (GIP Seine-Aval database); ^b^cumulated rainfall 7 days before the sampling (Météo France database); ^c^Suspended Particulate Matter (wet weight); ^d^CFU.100 mL^-*1*^; ^e^CFU.g^-*1*^ (wet weight); ^f^count below the detection threshold; NA: not analyzed; NS: not sampled.*

#### *Eurytemora affinis* Sampling

Living copepods were collected in the subsurface using a WP2 plankton net (200-mm mesh size; 1 m in diameter) as previously described (Cailleaud et al., 2007). Immediately after sampling, copepods were sorted using two sieves (500- and 100-μm mesh size) in order to eliminate particles and predators such as *Mysidacea* and *Gammaridae.* Copepods were then stored in insulated containers filled with estuarine water and microbiological analysis was carried out within 4 h.

#### Chemical and Physical Parameters

Temperature, salinity, and conductivity were measured using a *in situ* multi-parameter probe (TetraCon 325, WTW, Germany). To determine the SPM concentration, the water was filtered through preweighed 0.45-μm pore-size filters (Whatman GF/F, Sigma–Aldrich). After filtration, the filters were rinsed with distilled water (10% filtered volume) to remove the salt and dried for 48 h at 50°C before being weighed again to determine the total SPM concentration. Rainfall was extracted from Méteo-France database^2^. River flow was extracted from the GIP-SA database^3^, as was the flow rate.

### Enumeration and Isolation of Culturable *E. coli*, *Enterococcus*, and Presumptive *Aeromonas*

*Escherichia coli*, *Enterococcus*, and presumptive *Aeromonas* were enumerated using membrane filtration methods (ACN, 0.45 μm, Sartorius). β-D-galactosidase- and β-D-glucuronidase-positive *E. coli* were isolated from the water samples with selective chromogenic media specific for *E. coli*, with the addition of a selective supplement for water samples (RAPID’*E. coli* 2 Medium and Supplement; Biorad, United States); plates were incubated for 24 h at 37°C. *Enterococcus* was isolated from the water samples with selective chromogenic media specific for *Enterococcus* (RAPID’*Enterococcus* Medium; Biorad, France); the plates were incubated for 48 h at 44°C. Presumptive *Aeromonas* were isolated on *Aeromonas* isolation agar based on Ryan’s formulation (Sigma–Aldrich, United States) supplemented with ampicillin (5 mg.L^-1^) (Sigma–Aldrich, United States). Plates were incubated for 48 h at 22°C. Dark green colonies (sulfide production and no acid formation) were considered as presumptive *Aeromonas* until molecular characterization, as described in Section Antimicrobial Susceptibility Testing. The sediment was analyzed with the following modifications: 3 g (w/w) were added to 27 mL of NaCl 0.85% (w/v) supplemented with Na_4_P_2_O_7_ (1 mM, final concentration) and mixed vigorously for 3 min to dissociate bacteria from organic mineral particles. Ten-milliliter volumes of appropriate dilutions were then filtered (ACN, 0.45 μm, Sartorius) before plating. Further identification of presumptive *Aeromonas* (*rad*A^-^) was carried out using mass spectrometry (matrix-assisted laser desorption ionization mass spectrometry-time of flight, MALDI-TOF MS); Biotyper (Bruker Daltonics, Germany).

Copepods previously collected in estuarine water were separated from suspended particles by phototropism (915 lumens) for 15 min, then sorted using a 200-μm sieve, resuspended in 5 L of artificial brackish water (15 PSU), and filtered on a 1.2-mm filter (Sartorius, France) before being weighed. Finally, copepods were suspended in 30 mL of NaCl 0.9% (w/v) and mixed with a blender (Ultra Turrax T10, Imlab, France) for 1 min at 4°C before being analyzed. Ten-milliliter volumes of appropriate dilutions were then filtered before plating (0.45 μm HA047, Millipore). The threshold values for the enumeration of *Enterococcus*, *E. coli*, and presumptive *Aeromonas* in water was 5 CFUs per 100 mL. For each sample, non-confluent colonies of presumptive *Aeromonas* were selected on the filter and then streaked on Luria Broth agar (Thermo Fisher Scientific). Finally, a total of 476 presumptive *Aeromonas* strains (dark green colonies) were isolated and stored on a cryo-bead system (Dutscher) at -80°C.

### Antimicrobial Susceptibility Testing

*Aeromonas* resistance to antibiotics was tested using the disk diffusion method according to the recommendations of the European Committee on Antimicrobial Susceptibility Testing (EUCAST) guidelines V1.0 2015. The categorical interpretations (susceptible, S; intermediate, I; resistant, R) were based on the EUCAST interpretative criteria for *Enterobacteriaceae* after incubation at 22 and 35°C for 24 h. *E. coli* CIP 7624 (ATCC 25922) was used as a control. The antibiotics tested (16) included the most commonly used in France for treatment of *Aeromonas* infections in human and veterinary medicine: ampicillin (AM, 10 μg), amoxicillin + clavulanic acid (AMC, 20 + 10 μg), ticarcillin (TIC, 75 μg), ticarcillin + clavulanic acid (TCC, 75 + 10 μg), piperacillin (PRL, 30 μg), piperacillin + tazobactam (TBZ, 30 + 6 μg), cefotaxime (CTX, 5 μg), cefoxitin (FOX, 30 μg), cefepime (FEP, 30 μg), ertapenem (ETP, 10 μg), imipenem (IPM, 10 μg), gentamicin (CN, 10 μg), tobramycin (TOB, 10 μg), norfloxacin (NOR, 10 μg), ciprofloxacin (CIP, 5 μg), trimethoprim + sulfamethoxazole (SXT, 23.75 + 1.25 μg). As recommended by Magiorakos et al. (2012), *Aeromonas* strains resistant to at least one antibiotic in three or more antimicrobial classes (penicillins not included) were considered a multidrug-resistant strain.

### DNA Extraction and PCR Amplification

Cell suspensions were prepared with two or three colonies in 200 μL of sterile water and total bacterial DNA was extracted by boiling (10 min at 94°C). All presumptive *Aeromonas* strains were tested and identified at the species level by amplification and sequencing of housekeeping genes. PCRs were performed with specific primers targeting the *gyrB* gene (Yáñez et al., 2003) and the *radA* gene (Roger et al., 2012), as previously described.

The PCR products were separated in 1.5% agarose gel in 0.5× TBE buffer. The products were sequenced using forward amplification primers in an ABI 3730XL automatic sequencer (Beckman Coulter Genomics). Membership in the genus *Aeromonas* was checked by comparison with the NCBI database using the Basic Local Alignment Search Tool (BLAST^4^). All the sequencing data were submitted to the GenBank database: the accession numbers of the sequences are KX898587 to KX898810.

### Phylogenetic Analysis

Phylogenetic analysis was performed as previously described by Roger et al. (2012). Briefly, gene sequences were codon-aligned using the ClustalW application within the Bioedit Sequence Alignment Editor. Phylogenetic analyses were performed for each of the two gene sequences and for a manually concatenated sequence. Gaps in concatenated sequences were deleted with Bioedit. The sequences were converted to Phylip format using the EMBOSS Seqret online program^5^. A maximum likelihood (ML) method-based phylogenetic tree was reconstructed using evolutionary distance analyzed with the PhyML v3.1 software using GTR, with a gamma distribution parameter estimated from the dataset and invariant sites as a substitution model. ML bootstrap support was calculated after 100 reiterations. Type strain sequences were downloaded from the NCBI database.

### Statistical Analysis

The chi-squared test of the Fisher exact test was performed to compare the antimicrobial profiles; the Pearson coefficient was used to measure the degree of linear correlation between abundance of *Aeromonas* in water and fluid mud; the Student’s *t*-test was used to compare the abundance of *Aeromonas* inside the water column. All data analyses were performed with XLSTAT (XLSTAT, Boston, MA, United States V2016.3).

## Results

### Abundance of *Aeromonas* in the Water Column and Copepods in the Estuarine Environment

In the oligohaline area of the Seine estuary, *Aeromonas* was detected in the water column, (i.e., surface water and fluid mud of the estuary mouth) for all flow rates and for water temperatures ranging from 5.4 to 19.8°C (**Tables 1**, **2A**). Copepods were collected in water characterized by a temperature varying between 7 and 19.5°C and a salinity between 0.6 and 5.2 (**Table 1**). Copepods were collected during campaigns (February 2012, August 2014, and October 2015) corresponding to low-flow periods when the HTZ was located in this area. Copepods were also detected during an increase of the river flow (March 2013), but not when the river flow reached 1000 m^3^s^-1^ (May 2013). For all of these campaigns, *Aeromonas* was always bound to copepods (**Table 2B**).

**Table 2A:** Occurrence of presumptive *Aeromonas* in water column.

	February 2012	March 2013	May 2013	August 2014	February 2015	June 2015	October 2015	November 2015
**Surface water (CFU.100 mL^-1^)**					
Presumptive *Aeromonas* spp.	1.1 × 10^2^	6.0 ± 0.2 × 10^2^	6.1 ± 1.3 × 10^2^	1.4 ± 0.5 × 10^2^	3.0 ± 2.8 × 10^2^	4.6 ± 0.3 × 10^2^	1.2 ± 0.3 × 10^3^	1.3 ± 0.6 × 10^2^
**Fluid mud (CFU.g^-1^)**					
Presumptive *Aeromonas* spp.	NA	1.2 ± 0.3 × 10^3^	5.7 ± 0.7 × 10^3^	9.0 ± 2 × 10^2^	5.3 ± 0.9 × 10^2^	9.1 ± 0.3 × 10^2^	9.8 ± 0.9 × 10^3^	2.6 ± 2.6 × 10^2^


*NA, not analyzed.*

**Table 2B:** Occurrence of presumptive *Aeromonas* and fecal indicator bacteria associated with *Eurytemora affinis* in Seine estuary.

	February 2012	March 2013	October 2015
**Presumptive *Aeromonas*^a^ (CFU.g^-1∗^)**	1.9 ± 0.7 × 10^2^	>1.1 × 10^4^	4.3 ± 0.6 × 10^3^
**Fecal indicator bacteria (CFU.g^-1∗^)**			
*E. coli*	3.6 ± 0.3 × 10^1^	7.9 ± 0.5 × 10^1^	1.0 ± 0.1 × 10^2^
*Enterococci*	6.4 ± 1.6 × 10^1^	2.9 ± 0.1 × 10^2^	1.8 ± 0.4 × 10^2^


*^∗^Wet weight copepods. ^a^Presumptive abundance based on phenotypic feature of *Aeromonas* on Aeromonas Isolation Agar (48 h at 22°C, sulfide production and no acid formation).*

In surface water, the abundance of presumptive *Aeromonas* (i.e., dark green colonies with sulfide formation and no acid formation) ranged from 1.1 × 10^2^ to 1.2 ± 0.3 × 10^3^ CFU.100 mL^-1^ and was not significantly different from the abundance in the bottom of the water column (*P*-value = 0.24) (**Table 2A**). In fluid mud, the abundance ranged from 2.6 ± 2.6 × 10^2^ to 9.8 ± 0.9 × 10^3^ CFU.g^-1^ (w/w). For each campaign, the abundance of presumptive *Aeromonas* in water and fluid mud were positively correlated (*R*^2^ = 0.8, *P*-value = 0.006). The abundance of presumptive *Aeromonas* associated with living copepods (see Materials and Methods) ranged from 1.9 ± 0.7 × 10^2^ to >1.1 × 10^4^ CFU.g^-1^ (w/w) (**Table 2B**).

During all sampling campaigns, the microbiological quality of the estuarine water estimated by the abundance of *E. coli* and *Enterococci* was of good to average quality according to the French water index (SEQ values, 2 × 10^2^ CFU 100 mL^-1^ to 2 × 10^3^ CFU 100 mL^-1^, for *E. coli*, and 2 × 10^2^ CFU 100 mL^-1^ to 10^3^ CFU 100 mL^-1^ for *Enterococcus*) established by the French Ministry of the Environment and Regional Water Agencies as well the WHO recommendations (WHO, 2011; **Table 1**). However, no correlation was observed between the abundance of presumptive *Aeromonas* and indicators of fecal bacteria in water, fluid mud, and copepods. The abundance of presumptive *Aeromonas* was always about one or two orders of magnitude higher than the abundance of *E. coli* and *Enterococci*.

Among the 476 isolates of presumptive *Aeromonas* that were collected, 213 strains were confirmed as belonging to the *Aeromonas* genus through partial sequencing of *gyrB* (169 strains) and/or *radA* (173 strains). Interestingly, the occurrence of *Aeromonas* (*gyr B*+ and/or *radA* +) seems higher in sediment and associated with copepods than in water (**Table 3**). Identification of a random sample of 50 isolates of presumptive *Aeromonas* (*radA*^-^) was further analyzed based on matrix-assisted laser desorption ionization time-of-flight mass spectrometry (MALDI-TOF MS). Among them, 29 isolates belonging to *Pseudomonas* spp., 11 isolates of *Serratia marcescens*, and eight isolates could not be identified by this approach. Interestingly, four isolates were detected as *Aeromonas* spp. by MALDI-TOF MS.

**Table 3:** Occurrence of *Aeromonas* among presumptive *Aeromonas* isolated in Seine estuary.

		Water	Fluid mud		Copepods		WWTP	
Presumptive *Aeromonas* abundance^a^								
(CFU.100 mL^-1^ or CFU.g^-1^)		6.5 ± 5.3 × 10^2^	4.0 ± 5.0 × 10^3^		7.6 ± 4.7 × 10^3^		5.3 ± 0.3 × 10^4^	
Isolated number		136	130		125		100	
*Aeromonas gyrB*^+^ and/or *radA*^+^^b^		33%	47%		50%		46%	
(CFU.100 mL^-1^ or CFU. g^-1^)^c^		2.1 ± 1.7 × 10^2^	1.9 ± 2.4 × 10^3^		3.8 ± 2.3 × 10^3^		2.4 ± 0.1 × 10^4^	


*^a^Presumptive abundance based on phenotypic feature of *Aeromonas* on *Aeromonas* Isolation Agar (48 h at 22°C, sulfide production and no acid formation); ^b^number of *Aeromonas* (*gyrB*^+^*radA*^+^)/total number of presumptive *Aeromonas*; ^c^estimated abundance taking into account % of *Aeromonas* (*gyrB*+ *radA*+).*

### Diversity of *Aeromonas* Populations from the Water Column and Copepods in the Seine Estuary

**FIGURE 2:**
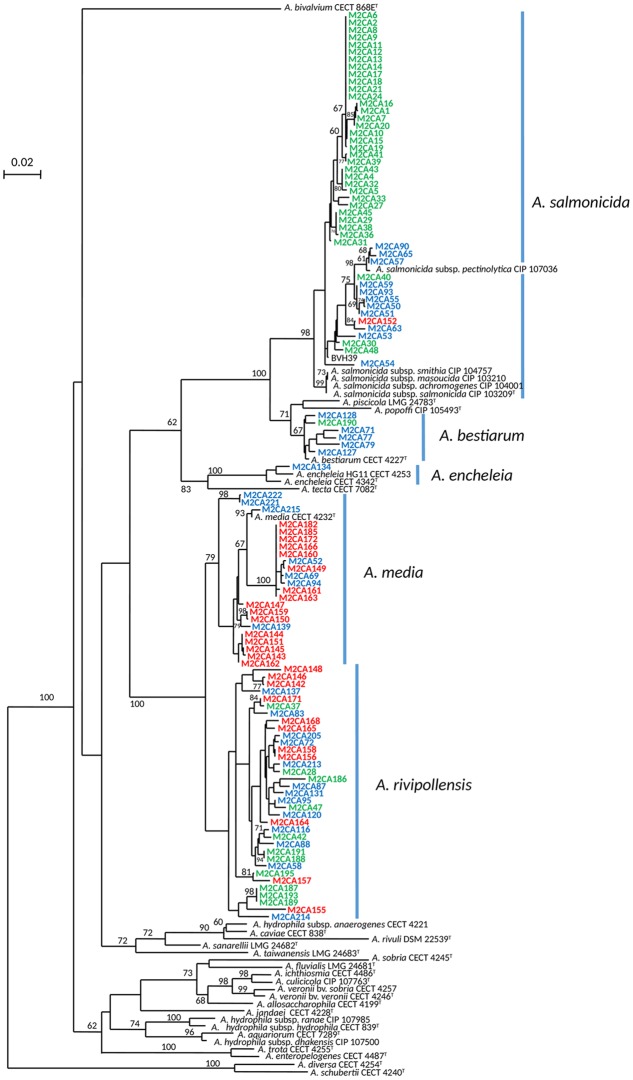
Unrooted maximum-likelihood tree based on concatenated sequences of the two housekeeping gene fragments (1182 nt). The tree shows the structure of the studied *Aeromonas* spp. population (110 strains). The origin of the trains is indicated by the font color, WWTP (red font), column water (blue font) and copepods (green font). The horizontal lines represent genetic distance, with the scale bar indicating the number of substitutions per nucleotide position. The numbers at the nodes are support values estimated with 100 bootstrap replicates. Only bootstrap values > 60 are indicated on the tree. Species names corresponding to the five clusters are indicated close to the blue bar.

**FIGURE 3:**
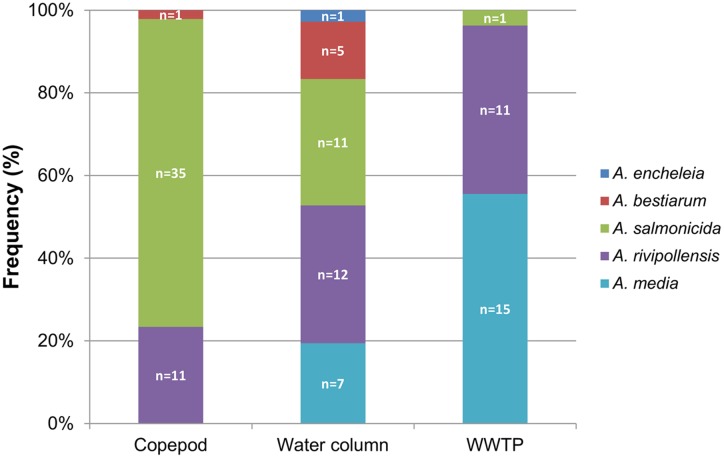
Distribution of *Aeromonas* species in the water column, associated with copepods, and in the effluent of WWTP. *n* = number of isolates.

To compare the diversity of the *Aeromonas* population sampled in the water column (surface water and fluid mud) with the *Aeromonas* population bound to the copepods, we studied all isolated *Aeromonas gyrB*^+^ and *radA*^+^, which comprised 47 isolates from copepods and 36 isolates from the water column. Phylogenetic analysis combined with phenotypic antibiotic-resistance profiles were carried out on all these isolates. In addition, 27 isolates of *Aeromonas* from WWTP-treated effluent (5.3 ± 0.3 × 10^4^ CFU.100 mL^-1^) located 1 km upstream from the sampling site were analyzed as a control corresponding to an input of allochthonous *Aeromonas* (i.e., not from an estuarine habitat). The phylogenetic tree reconstructed on the basis of concatenated sequences of *gyrB*+ *radA* (1182 nt) made it possible to discriminate the different *Aeromonas* species that were collected (**Figure 2**). Strains distributed in five clusters corresponding to *A. bestiarum* (*n* = 6; 5.45%), *A. encheleia* (*n* = 1; 0.91%), *A. media* (*n* = 22; 20.00%), and *A. salmonicida* (*n* = 47; 42.73%). The last cluster identified is phylogenetically very close to the *A. media* cluster and is probably affiliated with the recently described species *A. rivipollensis* (*n* = 34; 30.91%) (Marti and Balcázar, 2015) (**Figure 2**). In the water column, the five species were isolated as follows: *A. rivipollensis* (*n* = 12; 33.33%), *A. salmonicida* (*n* = 11; 30.56%), *A. media* (*n* = 7; 19.44%), *A. bestiarum* (*n* = 5; 13.89%), and *A. encheleia* (*n* = 1; 2.78%). Among the less common species, *A. bestiarum* was mainly isolated from the water column. While *A. salmonicida* is the major species that colonizes copepods, like more than 74.47% (*n* = 35) of *Aeromonas* strains, other species recovered include *A. rivipollensis* (*n* = 11; 23.40%) and *A. bestiarum* (only one strain). In contrast, in treated WWTP effluent, *A. media* (*n* = 15; 55.56%) and *A. rivipollensis* (*n* = 11; 40.74%) were mainly observed while only one strain of *A. salmonicida* was isolated (3.7%). It should be noted that *A. media*, a putative human pathogen, present in the water column and abundant in the WWTP samples, was not detected in association with living copepods (**Figures 2**, **3**).

**FIGURE 4:**
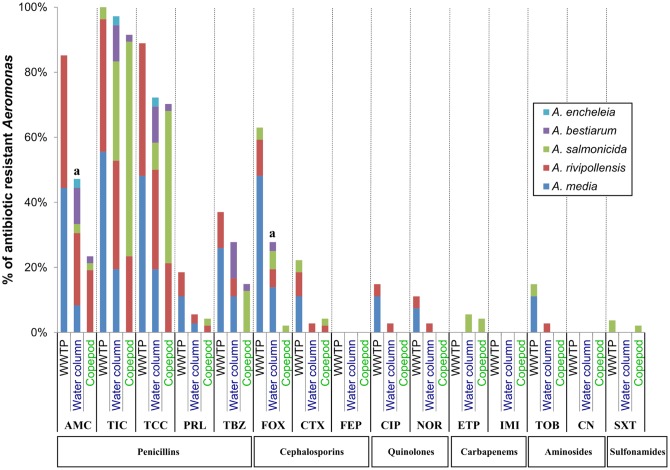
Comparative antibiotic resistance phenotypes of *Aeromonas* populations from water column, copepods and WWTP. The number (n) of isolates resistant to each of the 16 antibiotics was estimated using the agar diffusion method at 22°C, for *Aeromonas* populations sampled from the water column (*N* = 36); copepods (*N* = 47) and the effluent of WWTP (*N* = 27). Percentages of strains resistant to each antibiotic within *Aeromonas* population corresponded n/N. ^a^Significant difference between percentages of resistance to an antibiotic between the *Aeromonas* population isolated from the water column and copepods by the chi-squared test of Fischer (*P-*value = 0.02 for AMC and 0.001 for FOX). AM, Ampicillin; AMC, Amoxicillin + clavulanic acid; TIC, Ticarcillin; TCC, Ticarcillin + clavulanic acid; PRL, Piperacillin; TBZ, Piperacillin + tazobactam; CTX, Cefotaxime; FOX, Cefoxitin; FEP, Cefepime; ETP, Ertapenem; IMI, Imipenem; CN, Gentamicin; TOB, Tobramycin; NOR, Norfloxacin; CIP, Ciprofloxacin; SXT, Trimetropin + sulfamethoxazole.

Considering the profiles of antibiotic resistance phenotypes, no significant difference between *Aeromonas* (*gyrB*^+^ *radA*^+^) populations from the water column and copepods was observed, except for AMC (*P*-value = 0.02) and FOX (*P*-value = 0.001) resistance, which is higher in the *Aeromonas* population from the water column than in the *Aeromonas* population from copepods (**Figure 4**). However, at the species level, 62.9% of *A. salmonicida* associated with copepods were resistant to TCC versus 27.3% in the *Aeromonas* population from the water column (*P*-value = 0.04; Supplementary Table S1). No multiresistant *Aeromonas* was isolated from copepods. Only two multiresistant *Aeromonas* were isolated in the water column (*A. rivipollensis*) and in the WWTP effluent (*A. media*) (**Table 4**).

**Table 4:** Antibiotic-resistant phenotypes of *Aeromonas* (*gyrB*^+^ *radA*^+^) from Seine Estuary.

Origin of sample	Species	No. of isolates	No. of resistance	Pattern
**Copepod**	*A. salmonicida*	3	1	AM
		1	2	AM, TCC
		6	2	AM, TIC
		19	3	AM, TIC, TCC
		1	4	AM, TIC, TCC, ETP
		1	4	AM, AMC, TIC, TCC
		1	4	AM, TIC, TCC, TBZ
		1	4	AM, TIC, TCC, FOX
		1	5	AM, TIC, TCC, CTX, SXT
		1	6	AM, TIC, TCC, PRL, TBZ, ETP
	*A. rivipollensis*	1	3	AM, TIC, TCC
		9	4	AM, AMC, TIC, TCC
		1	4	AM, TIC, PRL, CTX
	*A. bestiarum*	1	5	AM, AMC, TIC, TCC, TBZ
**Water column^a^**	*A. salmonicida*	7	2	AM, TIC
		1	3	AM, TIC, FOX
		1	4	AM, AMC, TIC, TCC
		1	4	AM, TIC, TCC, ETP
		1	5	AM, TIC, TCC, FOX, ETP
	*A. rivipollensis*	1	2	AM, TIC
		2	3	AM, TIC, TCC
		6	4	AM, AMC, TIC, TCC
		1	5	AM, AMC, TIC, TCC, TBZ
		1	5	AM, AMC, TIC, TCC, FOX
		1^b^	10	AM, TIC, TCC, PRL, TBZ, CTX, FOX, TOB, NOR, CIP
	*A. bestiarum*	1	1	AM
		3	5	AM, AMC, TIC, TCC, TBZ
		1	6	AM, AMC, TIC, TCC, TBZ, FOX
	*A. media*	1	3	AM, TIC, TCC
		1	4	AM, TIC, TCC, FOX
		1	4	AM, TIC, TCC, TBZ
		1	5	AM, AMC, TIC, TCC, FOX
		2	6	AM, AMC, TIC, TCC, TBZ, FOX
		1	6	AM, TIC, TCC, PRL, TBZ, FOX
	*A. encheleia*	1	4	AM, AMC, TIC, TCC
**WWTP**	*A. salmonicida*	1	6	AM, TIC, CTX, FOX, TOB, SXT
	*A. rivipollensis*	4	4	AM, AMC, TIC, TCC
		3	5	AM, AMC, TIC, TCC, FOX
		1	5	AM, AMC, TIC, TCC, TBZ
		1	6	AM, AMC, TIC, TCC, NOR, CIP
		2	7	AM, AMC, TIC, TCC, PRL, TBZ, CTX
	*A. media*	1	2	AM, TIC
		1	4	AM, TIC, TCC, FOX
		5	5	AM, AMC, TIC, TCC, FOX
		1	6	AM, AMC, TIC, TCC, TBZ, FOX
		1	7	AM, AMC, TIC, TCC, PRL, TBZ, FOX
		1	7	AM, AMC, TIC, TCC, TBZ, CTX, FOX
		3	8	AM, AMC, TIC, TCC, PRL, TBZ, CTX, FOX
		2^b^	9	AM, AMC, TIC, TCC, TBZ, FOX, TOB, NOR, CIP


*^a^i.e., water + fluid mud; ^b^ strain resistant to at least three antibiotic classes (Penicillin group not included, as it is considered as an intrinsic resistance for *Aeromonas*, Baron et al., 2017); AM, Ampicillin; TIC, Ticarcillin; TCC, Ticarcillin + clavulanic acid; PRL, Piperacillin; AMC, Amoxicillin + clavulanic acid; TBZ, Piperacillin + tazobactam; CTX, Cefotaxime; FEP, Cefepime; FOX, Cefoxitin; CIP, Ciprofloxacin; NOR, Norfloxacin; ETP, Ertapenem; IMI, Imipenem; TOB, Tobramycin; CN, Gentamicin; SXT, Trimetropin + sulfamethoxazol.*

## Discussion

Aquatic environments including aquatic organisms are considered Aeromonad’s primary habitat (Janda and Abbott, 2010). Over the last decade, molecular approaches have greatly enhanced the knowledge of *Aeromonas* diversity in coastal or estuarine waters (Gugliandolo et al., 2008; Silva et al., 2014). Here, we confirm that *Aeromonas* should be identified using molecular methods for a better understanding of the diversity of *Aeromonas* in the oligohaline zone of a highly anthropized estuary (Seine). In this environment, only 44.7% of the presumptive *Aeromonas*, i.e., able to grow on a selective medium, were identified as *Aeromonas* based on the sequence of *rad*A and/or *gyr*B genes. Further identification using mass spectrometry (MALDI-TOF) shows that presumptive *Aeromonas (radA*^-^) could be mainly related to *Pseudomonas* and *Serratia.* These strains have the same culturable characteristics than *Aeromonas* on the selective media used in this study (*Aeromonas* Isolation Agar). However, mass spectrometry is not yet the most accurate method to identify all *Aeromonas* species or environmental bacteria (Shin et al., 2015). In this environment contaminated by metals, it has been shown that prevalence of *Pseudomonas* and *Enterobacteriaceae* as well as *Aeromonas* is high (Henriques et al., 2006). These results confirmed that housekeeping gene sequencing is the most accurate method to identify *Aeromonas* strains at the species level for strains recovered from aquatic environments, as described for aeromonads from other sources (Alperi et al., 2010b; Roger et al., 2012; Colston et al., 2014; Talagrand-Reboul et al., 2017).

Here we show that in the mouth of the macrotidal Seine estuary, *Aeromonas* spp. are detected in water and fluid mud during all hydrological periods. In contrast, in estuary water as well as in the coastal environment, a seasonal variation of *Aeromonas* abundance was reported. The maximum abundance of *Aeromonas* was observed in the urban area, during the high river flow period in a eutrophic tropical estuary (Abidjan, Senegal) and in spring in coastal waters (Messina, Italy) (Marcel et al., 2002; Silva et al., 2014). Moreover, in this study, the abundance of *Aeromonas* in water never exceeded 2 ± 1.7 × 10^2^ CFU.100 mL^-1^ and were lower than those reported in Italian and Brazilian anthropized estuaries (10^1^ to 10^6^ UFC 100 mL^-1^) (Gugliandolo et al., 2008; Silva et al., 2014). These discrepancies could be explained by (i) the methods used for counting *Aeromonas*, (ii) the impact of hydrosedimentary processes on *Aeromonas* behavior in a macrotidal estuary, (iii) the land use of the catchment basin that controls inputs of *Aeromonas* from soils, humans, and animals by surface runoff and waste waters, and (iv) the temperature of the Seine estuary water that is lower than the aeromonad optimal growth temperature of 22–25°C (Janda and Abbott, 2010). We show here that *Aeromonas* spp. are detected at the sediment–water interface of the fluid mud, which corresponds to the suspended particulate that settles at the bottom during slack water at high tide. Indeed, these results suggest that the behavior of *Aeromonas* in the mouth of the Seine estuary was strongly influenced by both the hydrology and particle dynamics (Guézennec et al., 1999; Malham et al., 2014). In the mouth of the Seine estuary, occurrence of *Aeromonas* is mainly related to the upstream input – i.e., treated WWTP effluent or watershed run off – as well as autochthonous *Aeromonas* able to grow in the oligohaline area of this highly anthropized environment. In addition, a secondary input of *Aeromonas* was related to the resuspension of surficial sediment, which previously settled on the intertidal mudflats located in this area (Berthe et al., 2008).

In the mouth of Seine estuary, *Aeromonas* spp. are also associated with *Eurytemora affinis* whose abundance depends on both their lifecycle and – as bacteria – the hydrosedimentological processes (Mouny and Dauvin, 2002; Devreker et al., 2010). Indeed, here we show that copepods are present when there is a HTZ in the oligohaline area of the Estuary. These results are consistent with a study reported by Devreker et al. (2010) that shows link between both salinity and the dynamic of SPM and those of copepods in the mouth of the Seine estuary. In similar estuarine water (e.g., the Adriatic Sea, Italy), it has also been shown that *Aeromonas* spp. were associated with copepods (*Temora stylifera*, *Acartia clausii*, *Centropages typicus*, and *Paracalanus parvus*) (Dumontet et al., 1996; Gugliandolo et al., 2008).

In this study, we show that the diversity of *Aeromonas* populations from the water column (water and fluid mud) is different from that associated with copepods. In the Seine estuary water column, five species of *Aeromonas* co-exist: *A. salmonicida*, *A. bestiarum*, *A. encheleia*, *A. media*, and *A. rivipollensis*. Indeed, such *Aeromonas* species could be well adapted to a estuarine environment having a cooler surface water with mean temperature of 13.4°C (min: 5.3°C/ max 21.3°C). Except for *A. salmonicida* (Henriques et al., 2006), these species, previously observed in farmed fishes (water and fish) (Esteve et al., 1995; Schmidt et al., 2001; Hatha et al., 2005; Behbahani et al., 2014; Bartkova et al., 2016) and in waste water (Figueira et al., 2011; Marti and Balcázar, 2015; Popovic et al., 2015; Vaz-Moreira et al., 2015; Varela et al., 2016), have never been, as far as we know, described in coastal and anthropized estuary waters. Only the species *A. hydrophila*, *A. caviae*, *A. sobria*, and *A. veronii* have been reported in coastal or estuarine environments (the Adriatic Sea, Italy) (Dumontet et al., 1996; Fiorentini et al., 1998; Gugliandolo et al., 2008). Underestimation of *Aeromonas* diversity could mainly stem from the identification methodology. However, both the sampling strategy and the water temperature could also explain the differences observed between the diversities of the *Aeromonas* population in these similar aquatic environments. Consequently, these factors deserve to be precisely reported in further studies. In this study, an input of *A. media* and *A. rivipollensis* from the treated influent of the closest WWTP cannot be excluded even if no correlation between abundance of *Aeromonas* and fecal bacterial indicators was observed. Indeed, in this anthropized estuary, the diversity of *Aeromonas* populations in the water column probably reflects the coexistence of autochthonous *Aeromonas* (*A. salmonicida*, *A. bestiarum*, *A. encheleia*), for which the estuary is the primary habitat, and *Aeromonas* previously released from wastewater. Interestingly, one of the dominant *Aeromonas* species from WWTP outflow in Monfort and Baleux’s (1990) study was *A. caviae*, which could possibly be wrongly identified because (i) this species is known to be difficult to phenotypically distinguish from *A. media* and *A. rivipollensis*, and (ii) *A. rivipollensis* was an unrecognized species at time of study.

In the Seine estuary, the *Aeromonas* population associated with *Eurytemora affinis* was composed of three species described today as non-human pathogens and was dominated by *A. salmonicida*. None of these species (*A. rivipollensis*, *A. salmonicida*, or *A. bestiarum*) has been previously described as being associated with copepods. To date, only the species *A. hydrophila* has been reported in association with copepods, probably due to the lack of resolution of the biochemical methods used in these studies (Dumontet et al., 1996; Gugliandolo et al., 2008). The higher aeromonad density associated with copepods may be due to the known aeromonad ability to produce biofilm and favor copepod colonization, and this should require further study. Interestingly, higher resistance to TCC was observed within the *A. salmonicida* population associated with copepods. As aeromonads are naturally resistant to Penicillins (Baron et al., 2017), these results suggest the existence of a specific population of *A. salmonicida* belonging to the microbiota of *Eurytemora affinis*, although further study is warranted to confirm this assumption.

## Conclusion

In the oligohaline zone of a highly anthropized estuary (oceanic climate), *Aeromonas* spp. (*gyrB*^+^ *radA*^+^) are present in the water column (water and fluid mud) and associated with living copepods (*Eurytemora affinis*). However, the diversity of the *Aeromonas* populations in the water column (water and fluid mud) is different from those associated with copepods. In the water column, the *Aeromonas* species autochthonous of the estuary, i.e., *A. salmonicida*, *A. bestiarum*, *A. encheleia*, co-exist with *Aeromonas* species (*A. media, A. rivipollensis*) originating from human and animal sources discharged by the WWTP effluent. *A. salmonicida* are the major species bound to the copepods (*Eurytemora affinis*), even if *A. rivipollensis* and *A. bestiarum* are also detected, while no human pathogenic species were associated with copepods. The proportion of *A. salmonicida* resistant to the antibiotic (TCC) was higher than in the *Aeromonas* population in the water column. These results underlined the key role played by this type of anthropized estuary, i.e., as an environment where autochthonous aeromonads and those originating from human and animal sources coexist.

## Author Contributions

FP, BL, and EJ-B defined the research theme. GC, TB, RL, JF-L, and FP defined sampling strategy and designed methods and experiments. GC carried out the laboratory experiments, strains isolation and antibiotic resistance analysis, FR carried out the phylogeny of aeromonads. GC, FR, TB, and FP analyzed and interpreted the data. FP and GC drafted the manuscript. BL, TB, RL, and JF-L revised the paper critically. All authors read and approved the final manuscript.

## Conflict of Interest Statement

The authors declare that the research was conducted in the absence of any commercial or financial relationships that could be construed as a potential conflict of interest.

## Footnotes

1 www.seine-aval.fr

2 www.meteociel.fr

3 http://www.seine-aval.fr/

4 http://blast.ncbi.nlm.nih.gov/Blast.cgi

5 http://www.ebi.ac.uk/Tools/sfc/emboss_seqret/
