# Supplementary material for: Distinct Aeromonas Populations in Water Column and Associated with Copepods from Estuarine Environment (Seine, France)
Source: Front Microbiol. 2017 Jul 11;8:1259. doi: 10.3389/fmicb.2017.01259 (PMC5504101; doi:10.3389/fmicb.2017.01259)
Supplement: Supplementary file 1 [file Table_1.PDF]

| | | *A. media* | *A. rivipollensis* | *A. salmonicida* | *A. bestiarum* | *A. encheleia* |
|---|---|---|---|---|---|---|
| **TIC** | Water column | 100.0% | 100.0% | 100.0% | 80.0% | 100.0% |
| | Copepods | nd | 100.0% | 88.6% | 100.0% | nd |
| | WWTP | 100.0% | 100.0% | 100.0% | nd | nd |
| **TCC** | Water column | 100.0% | 91.7% | 27.3% | 80.0% | 100.0% |
| | Copepods | nd | 90.9% | 62.9% | 100.0% | nd |
| | WWTP | 86.7% | 100.0% | 0.0% | nd | nd |
| **PRL** | Water column | 14.3% | 8.3% | 0.0% | 0.0% | 0.0% |
| | Copepods | nd | 9.1% | 2.9% | 0.0% | nd |
| | WWTP | 20.0% | 18.2% | 0.0% | nd | nd |
| **AMC** | Water column | 42.9% | 66.7% | 9.1% | 80.0% | 100.0% |
| | Copepods | nd | 81.8% | 2.9% | 100.0% | nd |
| | WWTP | 80.0% | 100.0% | 0.0% | nd | nd |
| **TBZ** | Water column | 57.1% | 16.7% | 0.0% | 80.0% | 0.0% |
| | Copepods | nd | 0.0% | 17.1% | 100.0% | nd |
| | WWTP | 46.7% | 27.3% | 0.0% | nd | nd |
| **CTX** | Water column | 0.0% | 8.3% | 0.0% | 0.0% | 0.0% |
| | Copepods | nd | 9.1% | 2.9% | 0.0% | nd |
| | WWTP | 20.0% | 18.2% | 100.0% | nd | nd |
| **FEP** | Water column | 0.0% | 0.0% | 0.0% | 0.0% | 0.0% |
| | Copepods | nd | 0.0% | 0.0% | 0.0% | nd |
| | WWTP | 0.0% | 0.0% | 0.0% | nd | nd |
| **FOX** | Water column | 71.4% | 16.7% | 18.2% | 20.0% | 0.0% |
| | Copepods | nd | 0.0% | 2.9% | 0.0% | nd |
| | WWTP | 86.7% | 27.3% | 100.0% | nd | nd |
| **CIP** | Water column | 0.0% | 8.3% | 0.0% | 0.0% | 0.0% |
| | Copepods | nd | 0.0% | 0.0% | 0.0% | nd |
| | WWTP | 20.0% | 9.1% | 0.0% | nd | nd |
| **NOR** | Water column | 0.0% | 8.3% | 0.0% | 0.0% | 0.0% |
| | Copepods | nd | 0.0% | 0.0% | 0.0% | nd |
| | WWTP | 13.3% | 9.1% | 0.0% | nd | nd |
| **ETP** | Water column | 0.0% | 0.0% | 18.2% | 0.0% | 0.0% |
| | Copepods | nd | 0.0% | 5.7% | 0.0% | nd |
| | WWTP | 0.0% | 0.0% | 0.0% | nd | nd |
| **IMI** | Water column | 0.0% | 0.0% | 0.0% | 0.0% | 0.0% |
| | Copepods | nd | 0.0% | 0.0% | 0.0% | nd |
| | WWTP | 0.0% | 0.0% | 0.0% | nd | nd |
| **TOB** | Water column | 0.0% | 8.3% | 0.0% | 0.0% | 0.0% |
| | Copepods | nd | 0.0% | 0.0% | 0.0% | nd |
| | WWTP | 20.0% | 0.0% | 100.0% | nd | nd |
| **CN** | Water column | 0.0% | 0.0% | 0.0% | 0.0% | 0.0% |
| | Copepods | nd | 0.0% | 0.0% | 0.0% | nd |
| | WWTP | 0.0% | 0.0% | 0.0% | nd | nd |
| **SXT** | Water column | 0.0% | 0.0% | 0.0% | 0.0% | 0.0% |
| | Copepods | nd | 0.0% | 2.9% | 0.0% | nd |
| | WWTP | 0.0% | 0.0% | 100.0% | nd | nd |

**Supplemental Table S1:** Percentages of *Aeromonas* isolates resistant to each antibiotic tested. Nd: not determined because no *Aeromonas* belonging to these species was isolated from the sample.
